# Supermicrosurgery Training Model: A Step-by-Step Illustrated Guide to the Chicken Thigh Free Flap

**DOI:** 10.7759/cureus.103174

**Published:** 2026-02-07

**Authors:** Carolina Chaves, Leonor Caixeiro, Filipa Poleri, Horácio Zenha, Horacio Costa

**Affiliations:** 1 Plastic, Reconstructive, Cranio-Maxillofacial and Hand Surgery and Microsurgery Unit, Unidade Local de Saúde Gaia/Espinho, Vila Nova de Gaia, PRT; 2 Microsurgery and Experimental Surgery Unit, Universidade de Aveiro, Aveiro, PRT

**Keywords:** chicken thigh free flap, microsurgery training, non-living model, plastic surgery, supermicrosurgery

## Abstract

Microsurgery is an essential skill of the modern plastic and reconstructive surgeon and one that requires practice to master. Consequently, cost-effective training models, preferentially those that spare living animal sacrifice, are crucial to training. The chicken thigh is an easily obtainable and affordable model that accurately mimics human vessels.

In this article, we present an illustrated step-by-step guide to harvest and anastomose a muscular free flap of the chicken thigh, using the puboischiofemoralis pars lateralis muscle. The smallest vessels dissected had a diameter of 0.8 mm, allowing supermicrosurgery training.

The puboischiofemoralis muscular flap can be consistently harvested, and its pedicle anastomosed to distal branches of the femoral vessels. The model provided a cost-effective simulation of vessel preparation and anastomosis suitable for microsurgical practice. The puboischiofemoralis complex muscular free flap of the chicken thigh is a reproducible, easily available training model for the practice of microsurgical and supermicrosurgical skills.

## Introduction

Microsurgery represents one of the most significant technical advancements in modern surgery [1]. Since the early 20^th^ century, the evolution of microsurgical techniques has been closely linked to key technological and conceptual milestones. Carrel’s description of the triangulation method for end-to-end vascular anastomosis in 1902 laid the technical foundation for modern microvascular surgery [2]. This was followed by the discovery of heparin in 1916, which enabled safer vascular manipulation [3], and the introduction of the surgical microscope into human surgery in the 1920s, significantly improving precision and outcomes [4]. The clinical relevance of these developments became evident in the 1960s, with landmark reports of limb and digit replantation, firmly establishing microsurgery as a viable clinical discipline [1,5].

Nowadays, microsurgery is a fundamental skill for the plastic and reconstructive surgeon, as well as several other surgical specialties like neurosurgery, orthopedics, and ophthalmology. As microsurgery expanded, so did the recognition that its successful practice depends on prolonged, structured, and repetitive training [6]. Unlike many other surgical skills, microsurgical competence is highly technique-dependent and unforgiving of error, requiring exceptional hand-eye coordination, depth perception, and fine motor control. Consequently, the development of effective training models has been a longstanding priority in surgical education. An ideal training model should allow progressive skill acquisition, accurately simulate tissue handling, be ethically acceptable, be readily available, and be cost-effective.

A stepwise approach to microsurgical training is widely advocated [7,8]. Early stages typically involve synthetic materials, such as silicone or latex tubes and surgical gloves, which are useful for learning basic instrument handling and knot tying [9, 10]. However, these models fail to replicate the elasticity, fragility, and layered structure of biologic tissues. At the other end of the spectrum, live animal models, most commonly rats, provide the highest degree of physiologic realism and remain the reference standard for advanced training. Nevertheless, their use is associated with ethical concerns, regulatory constraints, increased costs, and the need for specialized facilities and supervision. These limitations have stimulated growing interest in non-living biologic models that can bridge the gap between synthetic practice and live animal surgery [11].

Among these, the chicken thigh has emerged as one of the most accessible and versatile non-living microsurgical training models. First popularized as a training platform by Marsh et al. [12], the chicken thigh offers vessels of appropriate caliber, realistic tissue planes, and consistent anatomy. Several studies have validated its usefulness for microsurgical dissection and anastomosis of the main femoral neurovascular bundle, demonstrating improvements in technical performance. As a result, the chicken thigh has become widely adopted in microsurgical training curricula worldwide. [10,12-15].

Despite its proven value, most described applications of the chicken thigh model are limited to the main femoral vessels, which are relatively large in diameter and may not sufficiently challenge trainees beyond the introductory level. In parallel, the clinical relevance of supermicrosurgery, commonly defined as anastomosis of vessels measuring approximately 0.3 to 0.8 mm, has increased substantially, driven by procedures such as lymphatic-venous anastomosis, distal digital replantation, and perforator-based free tissue transfer [16,17]. This evolution highlights the need for training models that allow repetitive practice on smaller-caliber vessels without resorting to live animal sacrifice.

The objective of the present report is to expand the educational scope of the chicken thigh model by describing, in a detailed and illustrated manner, the harvest and transfer of a muscular free flap. The muscle suggested is the puboischiofemoralis, which has two components, a pars medialis and a pars lateralis. Its origin is in the ventrolateral ischium and pubis, and its insertion is the distal two-thirds of the femur. It has the function to extend the hip joint and to adduct the thigh [18].

By guiding trainees beyond the standard femoral vessel dissection toward smaller distal branches suitable for microvascular anastomosis, this model aims to simulate key steps of free tissue transfer while increasing technical difficulty. In doing so, we propose a reproducible, ethically responsible, and cost-effective non-living training exercise that enhances both microsurgical and supermicrosurgical skill acquisition.

## Technical report

**Figure 1:**
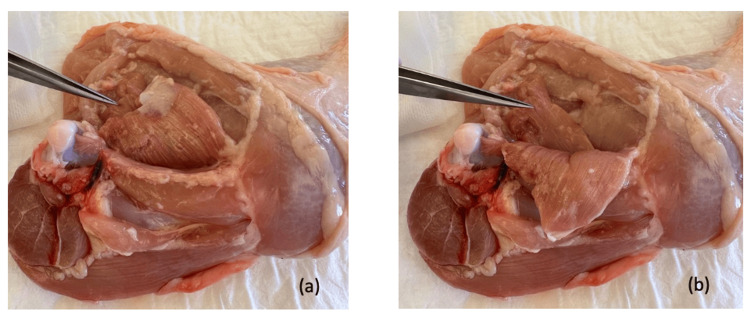
Chicken leg, medial view The puboischiofemoralis pars lateralis muscle was pointed out with microsurgical forceps (its proximal part was cut in the process of harvesting the chicken leg) before (a) and after (b) retraction of the puboischiofemoralis pars medialis muscle.

**Figure 2:**
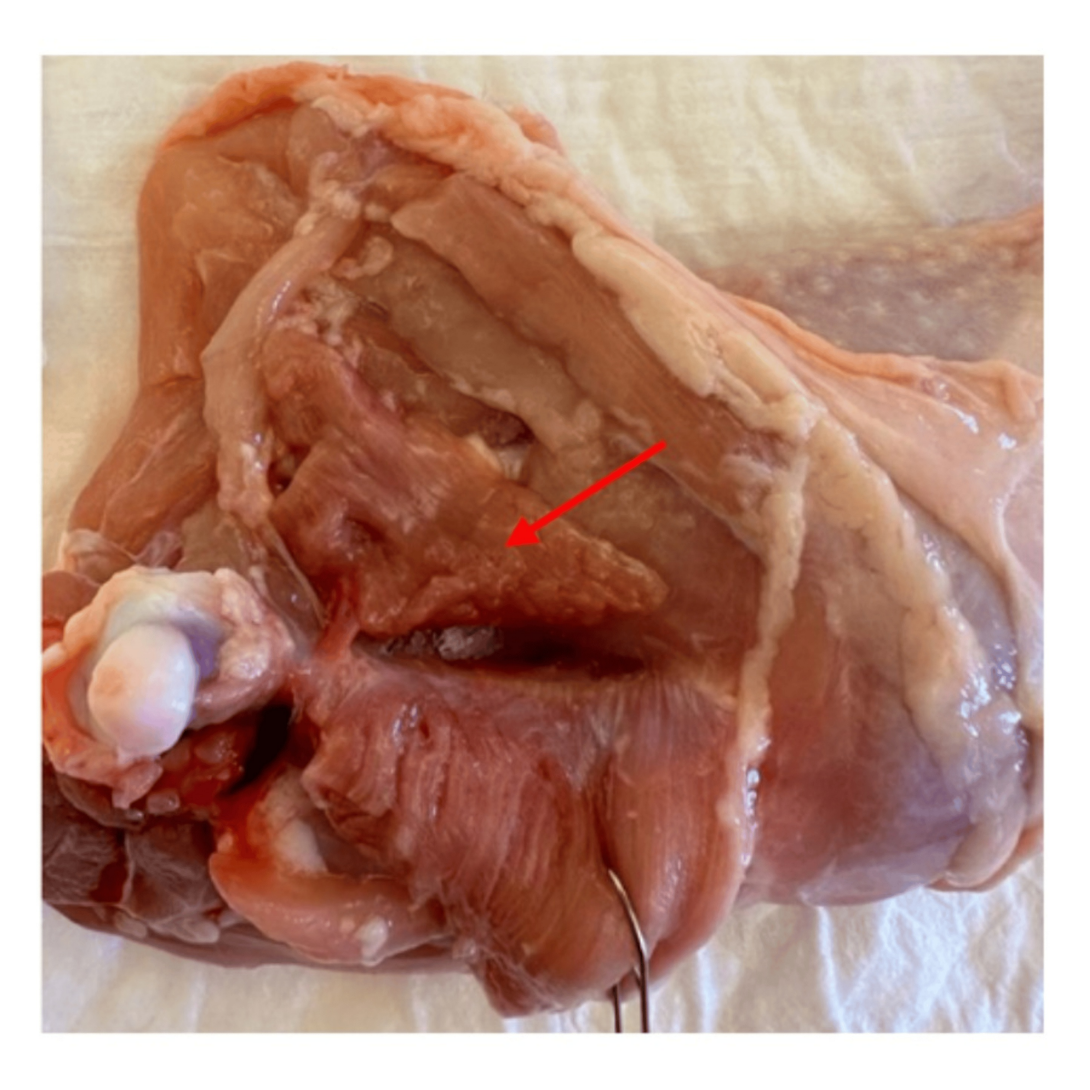
The puboischiofemoralis pars lateralis muscle after its disinsertion from the femur (red arrow). The puboischiofemoralis pars medialis muscle is retracted.

The exercise described in this report is the harvest of a muscular free flap of the chicken thigh and its anastomosis to more distal branches of the femoral artery and vein of the same chicken thigh. The muscle used was the puboischiofemoralis pars lateralis (Figures 1-2).

The approach was from the medial side of the thigh. The authors find this approach easier for reaching and dissecting the femoral neurovascular bundle. The leg was positioned as seen in Figure 1, with the more cephalic region of the thigh closer to the surgeon, during the entire dissection and anastomosis.

The skin was retracted and excised. Initial muscular dissection was preferentially blunt, using a round-pointed scissor, proceeding towards the femur until the nerve, artery, and femoral vein were visualized. A tip to find the neurovascular bundle is to orient the dissection in the direction of the femur, as the bundle is in the proximity of this bone and parallel to it. Using microsurgical forceps and scissors, the femoral nerve, artery, and vein were isolated. Dissection continued until the muscular branches of the femoral vessels supplying the puboischiofemoralis pars lateralis were identified. This initial phase was performed under a microscope magnification of six times.

The following step involved the separation of the muscular insertion of the puboischiofemoralis pars lateralis from the distal two-thirds of the femur (Figure 2). This separation must be done sharply, as this muscle is firmly attached to the distal two-thirds of the femur.

**Figure 3:**
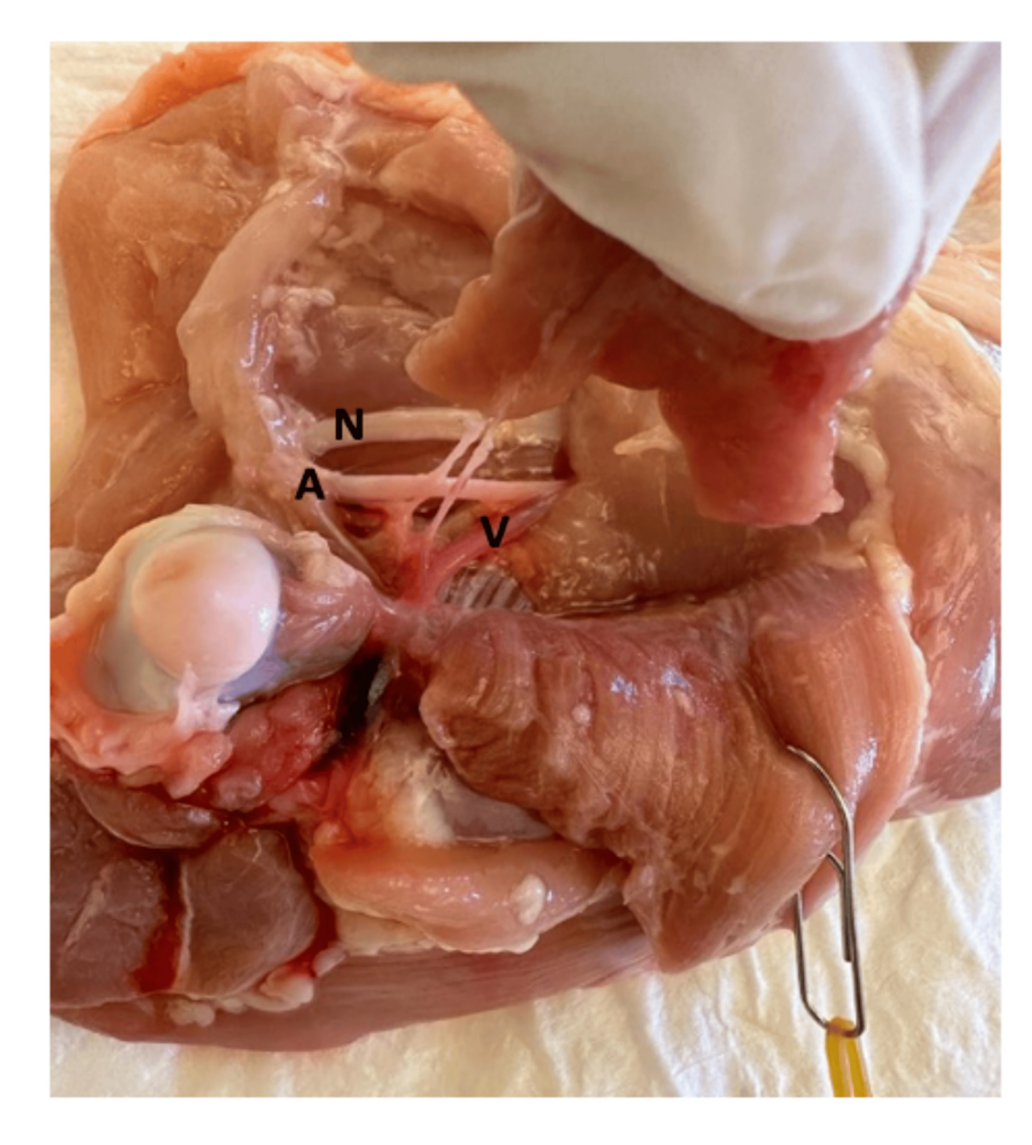
Puboischiofemoralis pars lateralis muscular flap fully dissected, attached only by its vascular pedicle. N: femoral nerve; A: femoral artery; V: femoral vein

**Figure 4:**
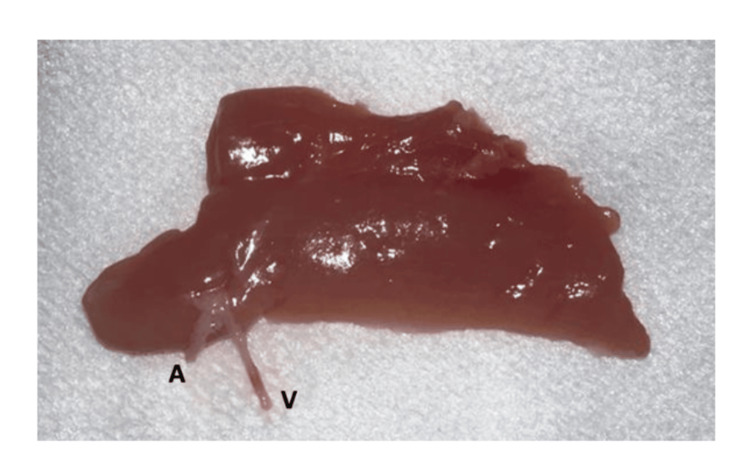
Puboischiofemoralis pars lateralis muscular flap separated A: artery; V: vein

The artery of the flap pedicle measured 1 mm in diameter, and the vein 0.8 mm. The dissection of the femoral artery and vein was continued distally until branches of similar caliber to the flap pedicle were found. These distal branches were dissected and cut as distally as possible (Figures 3-4).

**Figure 5:**
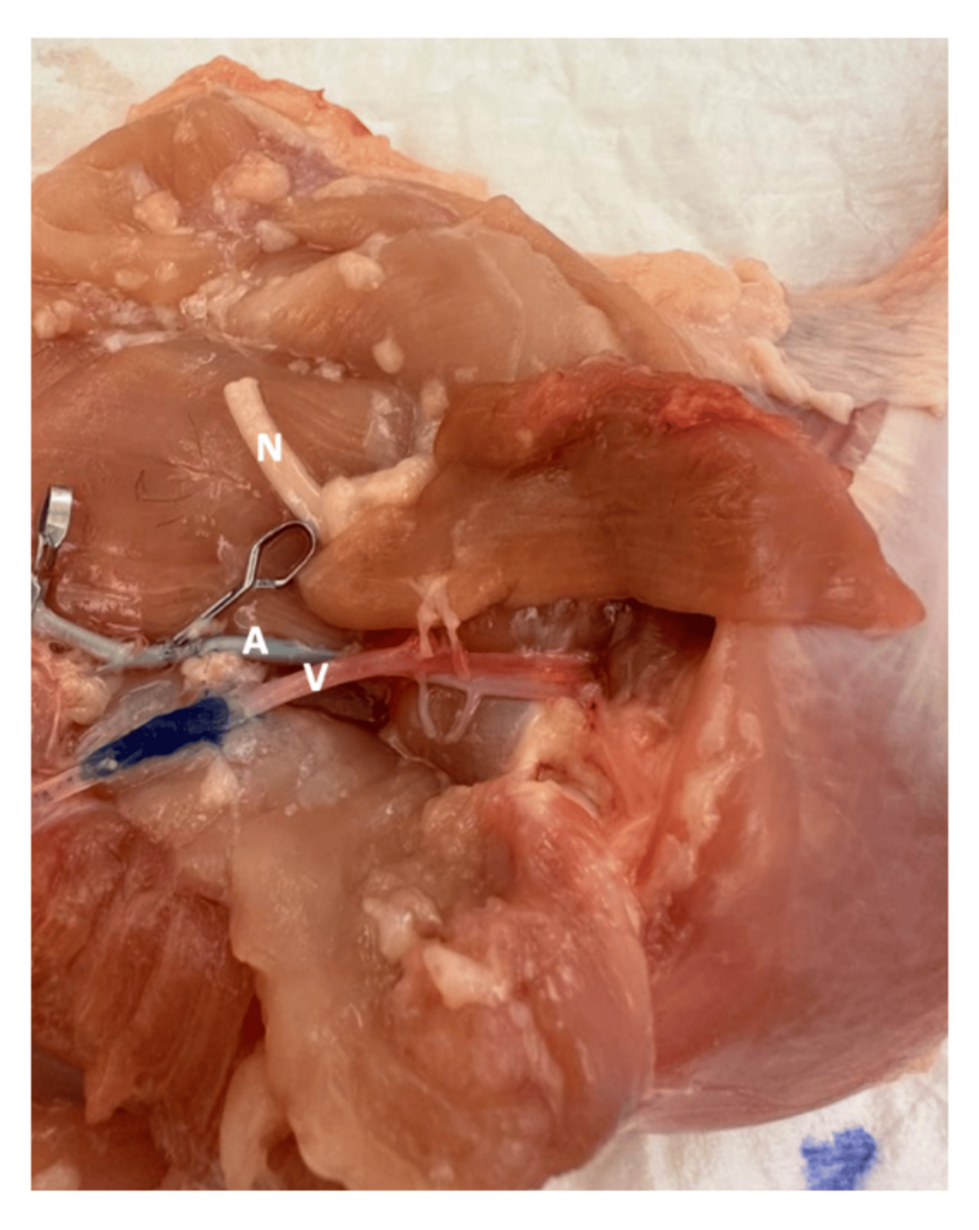
Flap positioned for anastomosis Distal branches of the femoral artery and vein are dissected and cut. Puboischiofemoralis pars lateralis muscle flap positioned for the vascular anastomosis. A: femoral artery; V: femoral vein; N: femoral nerve

The anastomosis of the flap pedicle to these branches of the main bundle was performed using a 10/0 nylon monofilament suture, in the position seen in Figure 5, in an end-to-end fashion, with interrupted sutures. The authors’ preferred technique is the use of two stay sutures, 180º apart, followed by suture of the anterior wall. Then the vessel is rotated, and the posterior wall is sutured. The number of sutures placed varies according to the vessel size. For this caliber (0.8 to 1 mm), six stitches are adequate: the two stay sutures plus two in the anterior and posterior wall. The suture used should be 10/0 or 11/0. This stage was conducted under a 10x to 16x magnification.

For this exercise, mainly for vessel dilation and anastomosis, the use of supermicrosurgery instruments (forceps tip between 0.05 and 1 mm) is advantageous. However, it is not a critical point, and this exercise and anastomosis can also be performed with normal microsurgery instruments. The anastomosis patency can be tested by injecting blue dye with a 30 or 32-G needle.

The expected timeframe for completion of this exercise is approximately one hour and 30 minutes. Still, with repeated practice, this time can be shortened.

## Discussion

Microsurgery is an essential skill for the modern reconstructive surgeon and one that requires a considerable amount of practice to master.

Although readily obtainable and noteworthy for the first contacts with microsurgery, the synthetic models lack resemblance to the biological tissues. [9] However, when jumping from these materials to the live animal training, there is a big gap in the proficiency needed. So, we consider the non-living model, like the chicken, a great in-between step [12-15]. To provide an even more gradual progression, after the usual dissection and anastomosis of the main vessels of the chicken thigh, we suggest the exercise described here of harvesting and anastomosing a muscular free flap of this anatomical area.

Before progressing to this exercise, it is advisable that the trainee has performed a few anastomoses of the artery and vein of the femoral vessels and has become familiar with the fragility of the 10/0 and 11/0 sutures.

The puboischiofemoralis pars lateralis was the chosen muscle for this exercise primarily because of its anatomical position. Its insertion in the distal two-thirds of the femur is close to the main neurovascular bundle, which allows the performance of the whole exercise without changing the thigh position [18]. Moreover, its harvest facilitates the exposure of the femoral vessels and the following steps of dissection.

To the best of our research, there is only one other article providing a guide to free flap harvesting in the chicken. Pafitanis et al. described the harvest of the adductor profundus muscle flap, part of the same complex of muscles of this article, but with a different nomenclature [19]. This variation arises because avian muscular anatomy is intricate, and its nomenclature can differ between sources. Our work complements this by offering an additional source of anatomical clarification, imagery, and orientation.

Although the complexity of this exercise is not comparable to the free flap harvest in a live model, the key step of performing the anastomosis of small-caliber vessels can be well simulated. The goal is to stimulate further dissection of the chicken thigh and vessels, past the commonly used main neurovascular femoral bundle. By targeting branches of smaller caliber, trainees are challenged with a higher level of difficulty. Since the venous pedicle in our model measured 0.8 mm in diameter, this exercise effectively serves as a training ground for supermicrosurgery [17, 20]. This makes the described exercise particularly relevant for trainees preparing for procedures such as distal digital replantation, perforator-to-perforator anastomosis, and lymphatic-venous anastomosis. [16].

Overall, this report reinforces the role of the chicken thigh as a versatile and scalable microsurgical training platform. By extending its use beyond basic exercises and into the realm of free flap harvest and supermicrosurgical-level anastomosis, this model fills an important gap in current training curricula. Its reproducibility, low cost, and ethical acceptability make it particularly well-suited for repeated practice and for training programs with limited access to live animal facilities.

## Conclusions

The chicken thigh is an extremely useful non-living model for microsurgery training. This report provides a guide to the harvest and transfer of a muscular flap of the chicken thigh by means of the puboischiofemoralis muscular complex. The goal is to encourage the dissection and anastomosis of smaller caliber vessels than the main neurovascular bundle of the avian thigh. This flap is reproducible and rewarding to perform, as it allows the trainee to touch the field of supermicrosurgery. In this manner, the usefulness of this easy-access and affordable model, which spares animal sacrifice, is increased.
